# Supplementary material for: Elevated fish densities extend kilometres from oil and gas platforms
Source: PLoS One. 2024 May 6;19(5):e0302738. doi: 10.1371/journal.pone.0302738 (PMC11073688; doi:10.1371/journal.pone.0302738)
Supplement: S1 Fig — Omnidirectional variograms of the residuals of the models fit for fish school presence/absence (a & b), fish school density (c &d) and individual fish (SED) density (e & f), before (a, c & e) and after (b, d & f) the implantation of data aggregation or correlation structure in the model. (DOCX) [file pone.0302738.s001.docx]

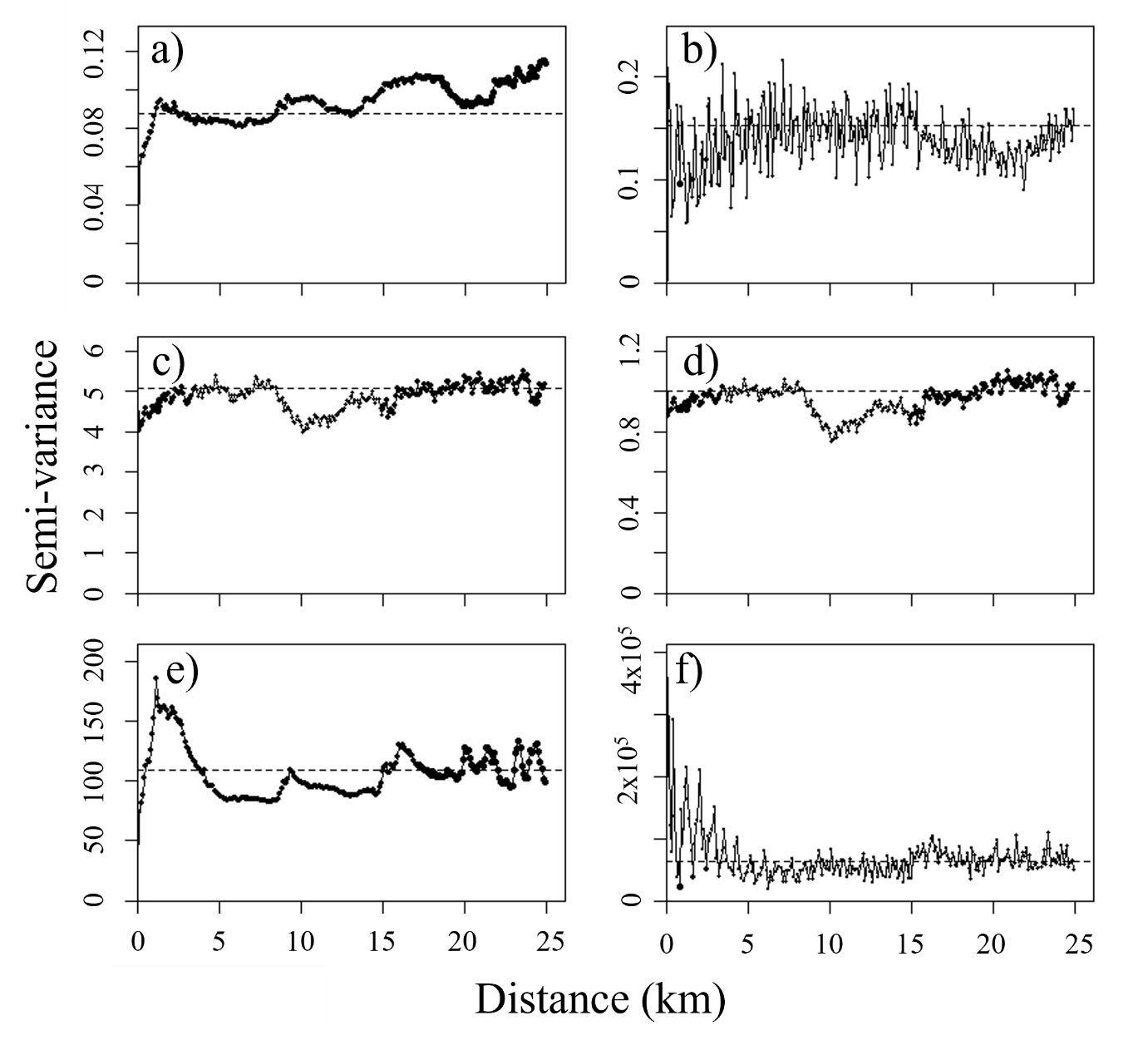


**S1 Fig. Residual variograms.** Omnidirectional variograms of the residuals of the models fit for fish school presence/absence (a & b), fish school density (c &d) and individual fish (SED) density (e & f), before (a, c & e) and after (b, d & f) the implementation of data aggregation or correlation structure in the model.
