# Supplementary material for: Elevated fish densities extend kilometres from oil and gas platforms
Source: PLoS One. 2024 May 6;19(5):e0302738. doi: 10.1371/journal.pone.0302738 (PMC11073688; doi:10.1371/journal.pone.0302738)
Supplement: S1 Table — Modelling results for factor variables from the GAM modelling fish school presence/absence, showing term estimates, standard errors, z- and p-values. The omitted factor levels (Platform Category: Fixed, ‘night’, ‘Bottom class 1’) are constituents of the model intercept. GBC abbreviates gravity-based concrete. (DOCX) [file pone.0302738.s002.docx]

| **Term** | **Estimate** | **Std. error** | **z-value** | **p-value** |
| --- | --- | --- | --- | --- |
| Platform category: Floating | -0.846 | 0.228 | -3.709 | 0.0002 |
| Platform category: GBC | -0.799 | 0.389 | 2.055 | 0.040 |
| Day | 1.420 | 0.186 | 7.621 | <0.0001 |
| Bottom class 2 | -0.755 | 0.399 | -1.894 | 0.058 |
| Bottom class 3 | -2.241 | 0.825 | -2.935 | 0.003 |
| Bottom class 4 | -1.072 | 0.421 | -2.546 | 0.011 |
| Bottom class 5 | -2.192 | 0.396 | -5.541 | <0.0001 |
| Bottom class 6 | -41.210 | 3.3 x 10^7^ | 0.000 | 1.000 |
| Bottom class 7 | 0.795 | 0.443 | 1.796 | 0.073 |
| Bottom class 8 | -0.258 | 0.488 | -0.529 | 0.597 |

**S1 Table. Modelling results for factor variables in the model of fish school presence/absence**

S1 Table. Modelling results for factor variables from the GAM modelling fish school presence/absence, showing term estimates, standard errors, z- and p-values. The omitted factor levels (Platform Category: Fixed, ‘night’, ‘Bottom class 1’) are constituents of the model intercept. GBC abbreviates gravity-based concrete.
