# Supplementary material for: Elevated fish densities extend kilometres from oil and gas platforms
Source: PLoS One. 2024 May 6;19(5):e0302738. doi: 10.1371/journal.pone.0302738 (PMC11073688; doi:10.1371/journal.pone.0302738)
Supplement: S2 Table — Modelling results for factor variables from the GAM modelling fish school density, where present, showing term estimates, standard errors, t- and p-values. The omitted factor levels (Platform Category: Fixed, ‘night’, ‘Bottom class 1’) are constituents of the model intercept. GBC abbreviates gravity-based concrete. (DOCX) [file pone.0302738.s003.docx]

**S2 Table. Modelling results for factor variables in the model of fish school density, where present**

| **Term** | **Estimate** | **Std. error** | **t-value** | **p-value** |
| --- | --- | --- | --- | --- |
| Platform category: Floating | 0.194 | 0.197 | 0.986 | 0.324 |
| Platform category: GBC | 0.557 | 0.314 | 1.776 | 0.076 |
| Day | 0.581 | 0.169 | 3.433 | 0.0006 |
| Bottom class 2 | 0.160 | 0.369 | 0.434 | 0.664 |
| Bottom class 3 | 1.577 | 0.772 | 2.041 | 0.041 |
| Bottom class 4 | -0.847 | 0.382 | -2.218 | 0.027 |
| Bottom class 5 | -0.195 | 0.369 | -0.529 | 0.597 |
| Bottom class 6 | 2.147 | 1.598 | 1.344 | 0.179 |
| Bottom class 7 | 0.168 | 0.413 | 0.406 | 0.685 |
| Bottom class 8 | -0.649 | 0.406 | -1.599 | 0.110 |

S2 Table. Modelling results for factor variables from the GAM modelling fish school density, where present, showing term estimates, standard errors, t- and p-values. The omitted factor levels (Platform Category: Fixed, ‘night’, ‘Bottom class 1’) are constituents of the model intercept. GBC abbreviates gravity-based concrete.
