# Supplementary material for: Elevated fish densities extend kilometres from oil and gas platforms
Source: PLoS One. 2024 May 6;19(5):e0302738. doi: 10.1371/journal.pone.0302738 (PMC11073688; doi:10.1371/journal.pone.0302738)
Supplement: S3 Table — Modelling results for factor variables from the GAM modelling individual fish (SED) density, showing term estimates, standard errors, z- and p-values. The omitted factor levels (Platform Category: Fixed, ‘night’, ‘Bottom class 1’) are constituents of the model intercept. GBC abbreviates gravity-based concrete. (DOCX) [file pone.0302738.s004.docx]

**S3 Table. Modelling results for factor variables in the model of individual fish density**

| **Term** | **Estimate** | **Std. error** | **z-value** | **p-value** |
| --- | --- | --- | --- | --- |
| Platform category: Floating | -0.045 | 0.071 | -0.630 | 0.529 |
| Platform category: GBC | 0.015 | 0.126 | 0.116 | 0.908 |
| Day | 0.329 | 0.052 | 6.306 | <0.0001 |
| Bottom class 2 | -0.269 | 0.133 | -2.030 | 0.042 |
| Bottom class 3 | 0.659 | 0.244 | 2.702 | 0.007 |
| Bottom class 4 | 0.132 | 0.133 | 0.989 | 0.323 |
| Bottom class 5 | -1.071 | 0.135 | -7.947 | <0.0001 |
| Bottom class 6 | -0.685 | 0.417 | -1.643 | 0.100 |
| Bottom class 7 | -0.341 | 0.158 | -2.150 | 0.032 |
| Bottom class 8 | 0.005 | 0.153 | 0.031 | 0.975 |

S3 Table. Modelling results for factor variables from the GAM modelling individual fish (SED) density, showing term estimates, standard errors, z- and p-values. The omitted factor levels (Platform Category: Fixed, ‘night’, ‘Bottom class 1’) are constituents of the model intercept. GBC abbreviates gravity-based concrete.
